# The Shape of Native Plant Cellulose Microfibrils

**DOI:** 10.1038/s41598-018-32211-w

**Published:** 2018-09-18

**Authors:** James D. Kubicki, Hui Yang, Daisuke Sawada, Hugh O’Neill, Daniel Oehme, Daniel Cosgrove

**Affiliations:** 1 Department of Geological Sciences, University of Texas at El Paso, El Paso, TX USA; 2 Department of Biology, The Pennsylvania State University, University Park, PA USA; 3 Department of Bioproducts and Biosystems, School of Chemical Engineering, Aalto University, Espoo, Finland; 4 Neutron Scattering Division, Oak Ridge National Laboratory, Oak Ridge, USA

## Abstract

Determining the shape of plant cellulose microfibrils is critical for understanding plant cell wall molecular architecture and conversion of cellulose into biofuels. Only recently has it been determined that these cellulose microfibrils are composed of 18 cellulose chains rather than 36 polymers arranged in a diamond-shaped pattern. This study uses density functional theory calculations to model three possible habits for the 18-chain microfibril and compares the calculated energies, structures, ^13^C NMR chemical shifts and WAXS diffractograms of each to evaluate which shape is most probable. Each model is capable of reproducing experimentally-observed data to some extent, but based on relative theoretical energies and reasonable reproduction of all variables considered, a microfibril based on 5 layers in a 34443 arrangement is predicted to be the most probable. A habit based on a 234432 arrangement is slightly less favored, and a 6 × 3 arrangement is considered improbable.

## Introduction

Cellulose is one of the most important materials on Earth. As the foundation of plant cell walls, it has a critical role in the biosphere. Because it has evolved to provide strength and resist biodegradation, cellulose is commonly used in paper, clothing, and myriad materials. Efforts to convert cellulose into fermentable compounds have become commercialized recently in order to produce ethanol from recyclable feedstocks that minimize impact on the food supply.

Landmark papers by Nishiyama and coworkers^1–3^ utilized X-ray and neutron diffraction to determine the crystalline structure of cellulose. This structure was used as the basis for density functional theory (DFT) calculations that subsequently reproduced the infrared, Raman, NMR and sum-frequency generation spectra of cellulose^4,5^. However, much of the research on cellulose has been performed on bacterial and/or plant-extracted cellulose neither of which represents the state of plant cellulose *in muro*^6,7^. Consequently, recent studies on non-extracted cellulose have been critical in helping to define the native state of cellulose within plant cell walls^8–10^.

Although the atomic structure of cellulose has been characterized, an outstanding question is the shape of cellulose microfibrils in plant cell walls. For decades, the cellulose microfibril (CMF) was thought to be comprised of 36 chains of cellulose polymers (i.e., 1,4 β-linked glucan monomers) based on the observation of “rosettes” formed by the cellulose synthase complex (CSC)^11^. Furthermore, a diamond-shaped habit based upon cellulose polymer layers arranged in a 1 × 2 × 3 × 4 × 5 × 6 × 5 × 4 × 3 × 2 × 1 pattern was assumed to be the habit of this 36-polymer CMF (e.g., Matthews *et al*., 2012). This view of the CMF was challenged by papers based on wide-angle X-ray scattering (WAXS)^12,13^. Based on diameter arguments, the 36-polymer CMF was judged to be too large, so the CMF would have to be smaller. This conclusion has been justified recently by the work of Nixon *et al*.^14^, Hill *et al*.^15^ and Venu *et al*.^16^ who have demonstrated that the CSC rosette is most consistent with a hexamer of trimers. Assuming only one cellulose polymer can be extruded from each of the CesA enzymes in the CSC^17–19^, the CMF should be made of 18 cellulose polymer chains rather than 36.

The difference between 18 and 36 polymers in the fundamental plant CMF will be reflected in the habit of the CMF as well as the size. The shape of the CMF is far from purely academic because the surfaces displayed by the CMF will depend upon this arrangement of cellulose chains. In turn, the interactions of other plant cell wall components (i.e., water, hemicellulose, pectin, lignin) will depend upon the nature of the exposed CMF surfaces. Biodegradation mechanisms of the cellulose will also depend heavily upon the nature of the CMF surfaces present. Hence, this study sought to determine the most probably arrangement of cellulose chains within the CMF in order to use this crystal habit as a basis for future studies of plant cell wall architecture, mechanics and biochemistry.

Based upon success in modeling cellulose atomic structure with density functional theory (DFT) calculations^4,20,21^, we chose to use a similar methodology^4^ to predict the structure, energetics and spectroscopic properties of finite CMFs with various arrangements of 18 cellulose chains. The periodic DFT calculations in VASP were carried out in order to obtain atomic structures that could be used for comparison against observed X-ray and neutron diffraction studies^1,2^. Furthermore, a key prediction is the relative energies of each CMF habit. Although one cannot expect CMFs formed in plant cell walls to necessarily be at thermodynamic equilibrium, Iβ cellulose is known to be thermodynamically stable and only slightly higher in Gibbs free energy than the extracted cellulose II and III forms^22^. Our assumption is that it does not make biochemical sense for plants to produce a less thermodynamically stable form both in terms of assembly kinetics and in regards to ultimate strength and resistance to biodegradation.

To supplement the atomic structure and thermodynamic criteria, this study also calculates the WAXS diffractograms and the ^13^C NMR chemical shifts (δ^13^C) of the model CMFs. The WAXS diffractograms were simulated using the CMF models taken from the structures as energy-minimized in VASP using the program WAXSiS^23,24^. δ^13^C values were calculated with Gaussian 09^25^ using the same model structures terminated after 4 glucan monomers along the c-axis with methyl groups to avoid unsatisfied valence at the terminal O atoms^8^ and following methods used in previous studies^26^.

## Results and Discussion

**Figure 1:**
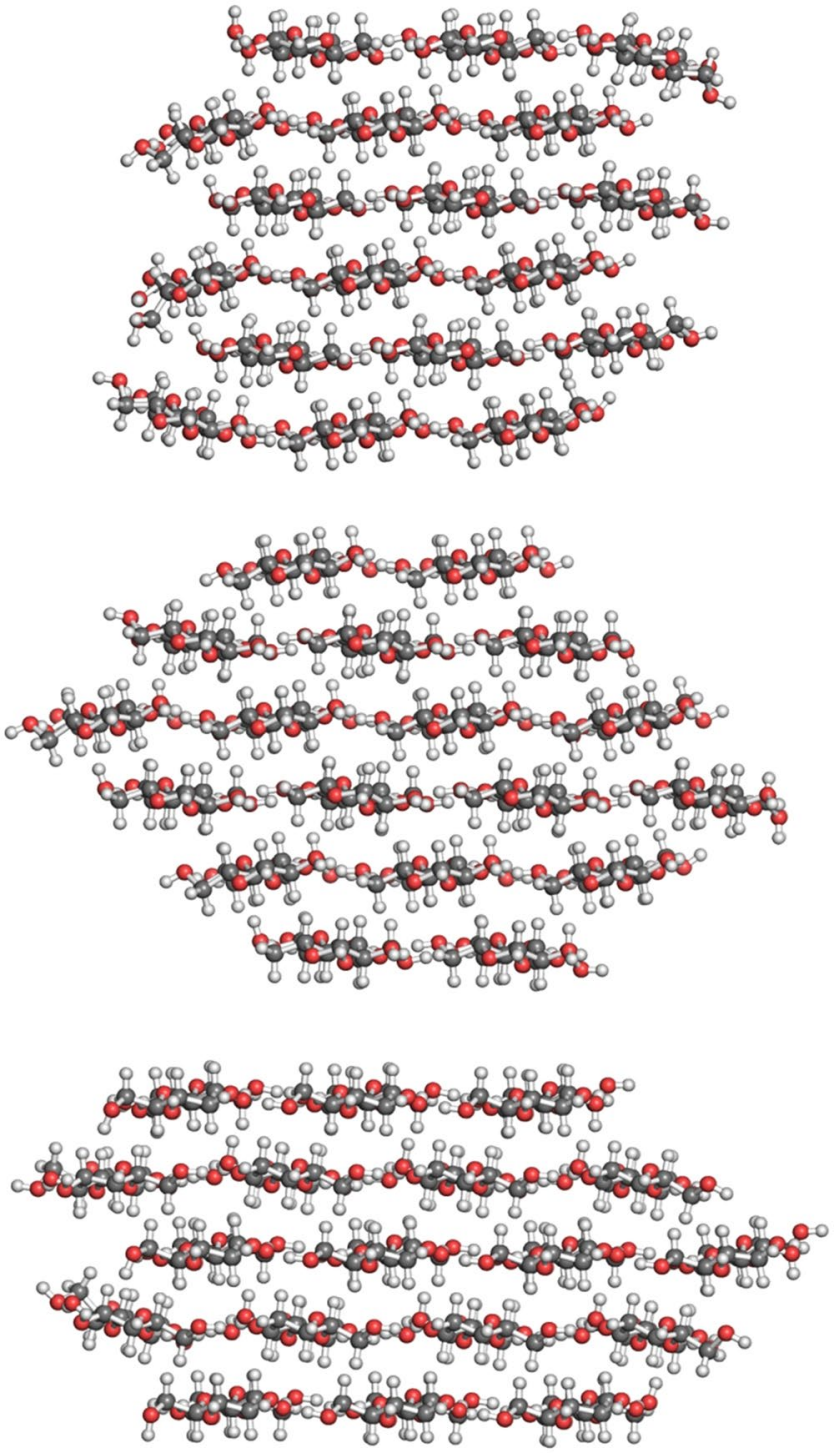
Potential crystal habits of native cellulose microfibrils: 6 × 3, 234432 and 34443 models (from top to bottom).

Three arrangements of the 18 cellulose polymers were created based upon the criteria that the CMF should attempt to minimize surface area, be reasonably symmetric and maximize the van der Waals forces between layers and H-bonding within layers. These three crystal habits are shown in Fig. 1. The model CMF habits have the following arrangements of cellulose polymers 6 × 3, 234432, and 34443. (A 3 × 6 structure is possible but the diameter of this shape is ≈5 nm – significantly wider than the 3 to 3.8 nm measured^12,13^. The first two have been suggested previously as shapes of the CMF (see ref.^13^), and the third is based upon a structure created for coarse-grained simulations of CMFs by Vin Crespi (The Pennsylvania State University).

Comparison of the model atomic structures with the parameters reported for Iβ cellulose in Nishiyama *et al*.^1,2^ reveals that all three CMFs accurately reproduce observed interatomic distances and torsion angles (Tables 1 and 2). Structural differences between the center and origin chains of Iβ cellulose are larger than the differences among the three CMF habits. Significant discrepancies exist between the model and experimentally-reported center chain H-bond distances and angles (Table 1), but we note that the model structures produce vibrational frequencies in agreement with observation^4,5^ and better agreement than the original Nishiyama *et al*.^1^ structure with observed δ^13^C values^4^. Because the same model and method reproduces the reported structure of the origin chain, we conclude that the discrepancy is not the result of inaccuracies in the model but instead originate from the difficulties arising from predicting H atom positions in cellulose^1^. The Φ (O5-C1-O4-C4), Ψ (C1-O4-C4-C5), χ (O5-C5-C6-O6) and χ′ (C4-C5-C6-O6) torsion angles calculated are in reasonable agreement with the experimental values (Table 2; refs^1,2^). There is no model that performs significantly better with respect to reproducing structural parameters, so we conclude that crystal habit has a minimal effect on these structural parameters.

**Table 1:** H-bonding and selected O–O distances (Å) and H-bond angles (O-H-O in degrees) in 6 × 3, 234432 and 34443 models.

	Origin				Center			
	Intra-molecular		intermolecular		Intra-molecular		intermolecular	
	O3H-O5	O2H-O6	O6H-O3	O6H-O2	O3H-O5	O2H-O6	O6H-O3	O6H-O2
Expt.	1.75 2.70 162	1.90 2.86 165	1.78 2.71 157	2.54 3.21 125	1.97 2.76 137	1.83 2.76 159	2.04 2.89 144	3.63 3.62 81
6 × 3	1.79 2.74 161	1.81 2.80 175	1.75 2.71 162	2.77 3.41 123	1.77 2.74 163	1.74 2.73 169	1.84 2.80 165	2.75 3.36 120
34443	1.79 2.74 161	1.82 2.81 174	1.74 2.70 163	2.77 3.39 121	1.77 2.74 163	1.74 2.73 169	1.83 2.80 163	2.75 3.36 121
234432	1.77 2.73 162	1.80 2.79 175	1.72 2.69 163	2.80 3.43 122	1.77 2.74 164	1.75 2.73 168	1.86 2.82 164	2.76 3.38 121

Experiment values are from refs^1,2^.

**Table 2:** Iβ cellulose torsion angles (degrees) in 6 × 3, 234432 and 34443 models.

	Origin				Center			
	Φ	Ψ	χ	χ′	Φ	Ψ	χ	χ′
Expt.	−88.7	−147.1	158.0	−83.0	−98.5	−142.3	170.0	−70.0
333333	−93.3	−146.1	168.9	−73.4	−90.3	−144.1	175.2	−67.1
34443	−93.2	−146.3	168.0	−74.2	−90.3	−144.1	176.2	−65.7
234432	−93.4	−146.5	170.6	−71.6	−90.0	−143.7	176.5	−65.0

Φ: O5C1O4C4, Ψ: C1O4C4C5, χ: O5C5C6O6, χ′: C4C5C6O6. Experiment values are from refs^1,2^.

Our second criterion for evaluating the probability of forming each CMF habit is the potential energy calculated for each. The periodic DFT calculations should better represent actual CMFs in that they are continuous rather than truncated after four glucan monomers. However, DFT has notable issues with estimating van der Waals forces^27^, so although the D3 dispersion correction of Grimme *et al*.^28^ was employed, this empirically-derived estimate is subject to a significant level of uncertainty. In order to test how robust the DFT-predicted energies might be, single-point energy calculations were also performed on the tetramer CMFs in Gaussian 09^25^ using the Perdew-Wang^29^, M05^30^, and B3LYP^31,32^ exchange-correlation functions in both vacuum and aqueous solution conditions (IEF-PCM)^33^. Table 3 lists the energies for each model and method. Both the VASP and Gaussian 09 results argue against the 6 × 3 model because it is higher in energy than the other two options. The +276 to +371 kJ discrepancy is large, but when normalized on a per glucan basis, the difference is 4 to 5 kJ/mol/glucan. However, this small energy difference is still not negligible, because an energy difference of 4 kJ/mol between two states would result in a Boltzmann distribution ratio of approximately 5 between the two states. The VASP energies indicate that the 234432 model is most stable whereas the Gaussian 09 calculations predict that the 34443 model is lower in energy in vacuum and aqueous solution. In the former case, the difference is less than 1 kJ/mol/glucan which is well within computational accuracy. Even the +4 kJ/mol/glucan difference favoring 34443 from Gaussian 09 is relatively small, so we cannot definitively distinguish between 234432 and 34443 based on energetics.

**Table 3:** Calculated energies of 6 × 3, 234432 and 34443 models with various methods (kJ/mol).

		34443	234432	6 × 3
*In vacuum*				
VASP	Rel. E	52	0	371
	Rel. E/glucan	0.7	0	5.1
M05-2X/6–31 G(d)	Rel. E	0	244.8	356.3
	Rel. E/glucan	0	3.4	4.9
B3LYP-D3/6–31 G(d)	Rel. E	0	260.4	435.8
	Rel. E/glucan	0	3.6	6.1
B3LYP/6–31 G(d)	Rel. E	0	335.6	291.4
	Rel. E/glucan	0	4.7	4.0
mpw1pw91/6–31 G(d)	Rel. E	0	316.6	276.6
	Rel. E/glucan	0	4.4	3.9
*In water (IEF-PCM)*				
M05-2X/6–31 G(d)	Rel. E	0	134.4	280.3
	Rel. E/glucan	0	1.9	3.9
B3LYP-D3/6–31 G(d)	Rel. E	0	155.0	363.9
	Rel. E/glucan	0	2.2	5.1
B3LYP/6–31 G(d)	Rel. E	0	230.3	219.6
	Rel. E/glucan	0	3.2	3.1

Rel. E: relative potential energy, Rel. E/glucan: relative potential energy normalized on a per glucan unit base. The lowest energy obtained from each method was set to 0 kJ/mol.

High-resolution ^13^C NMR spectra of cellulose *in vivo* have been reported recently^8^ that can be used as a basis for evaluating the proposed CMF habits. Table 4 compares observed and calculated values for all types of C atoms within the center and origin chains. For “interior” C atoms, all three model CMFs result in good agreement with observation with the 34443 model having a slightly better fit based on the root-mean-squared (RMSE) and maximum errors (MaxE). ^13^C NMR spectra of cellulose have observed C4, C5 and C6 peak doublets that have been ascribed to the changes in chemical environment and/or structure that occur on the surface of cellulose compared to the interior (see Harris *et al*.^9^, and references therein; Wang *et al*.^8^). This interior to exterior or ordered to disordered shift is especially pronounced for C4 which changes from 88.5 to 91.5 ppm on the interior (iC4) to 84 to 86 ppm on the surface (sC4). A similar shift may occur for C6 from iC6 of 66 ppm to sC6 of 62 ppm, but the sC6 peak is harder to resolve due to overlap with other plant cell wall polymers (Harris *et al*.^9^). Our results (Supplementary Table 1) indicate that surficial δ^13^C4 values (85.4 ± 0.9–87.5 ± 2.4 ppm) can match both the “iC4” and “sC4” peaks in Harris *et al*.^9^, and the surficial δ^13^C4 values are slightly higher than the interior δ^13^C4 values (83.9 ± 0.4–85.8 ± 0.6 ppm). Hence, the shift from 88.5 to 91.5 ppm to 84 to 86 ppm may not indicate interior versus exterior hydroxymethyl groups^34^. Furthermore, the shift is not necessarily the result of *tg* to *gt* rotations on the surface because none of the hydroxymethyl groups in our models are in a *gt* configuration. Instead, the difference may be caused by H-bonding or the lack thereof as well as other structural changes^34,35^.

**Table 4:** Observed and calculated ^13^C NMR chemical shifts (ppm) for interior cellulose chains.

	Observed		333333		34443		234432	
	c	b	Origin	Center	Origin	Center	Origin	Center
C1	104.1	104.8	106.0	102.8	105.4	102.5	105.2	102.4
C2	71.8	72.6	71.1	71.0	70.3	69.8	70.8	70.8
C3	75.2	75.4	72.5	72.7	73.2	73.9	72.8	72.6
C4	88.1	88.8	85.8	84.3	85.7	84.7	84.7	83.9
C5	71.2	72.6	72.4	71.6	73.0	70.9	72.5	71.4
C6	65.9	65.5	64.0	66.7	65.5	65.9	64.3	66.6
Slope			1.06	0.93	1.02	0.94	1.03	0.92
Intercept			−5.41	4.07	−2.23	2.59	−3.05	4.51
Correlation coefficient			0.993	0.993	0.993	0.996	0.992	0.993
MAE			1.8	2.2	1.6	2.1	1.8	2.4
RMSE			1.9	2.5	1.7	2.4	2.0	2.7
MaxE			2.7	4.5	2.4	4.1	3.4	4.9

Observed = Wang *et al*.^8^.

Ideal slope, intercept and correlation coefficient values are 1.0, 0.0 and 1.0, respectively. MAE = Mean Absolute Error, RMSE = Root-Mean-Squared Error, and MaxE = Maximum Error.

Wang *et al*.^36^ have found that some interior glucan chains in CMFs are more than one chain away from the nearest surface chains. Hence, the CMF of primary cell wall must have two types of interior cellulose chains: a core fraction not directly contacting with the surface and a bound fraction directly contacting with the surface. As shown in Supplementary Fig. 1, only the 34443 model has the core fraction, because chain A in 34443 is an interior chain, based on the calculated NMR chemical shifts as shown in Supplementary Table 2. 234432 and 333333 models do not have the core fraction. In addition, since chain A in 34443 is an interior chain, the ratio of surface chain and interior chain (s:i) for model 34443 is ~1.57, which is in good agreement with the s:i ratios of CMF in primary cell walls that Wang *et al*. found using solid-state NMR techniques. Therefore, based on the core domain and s:i ratio, the 34443 model is preferred.

**Figure 2:**
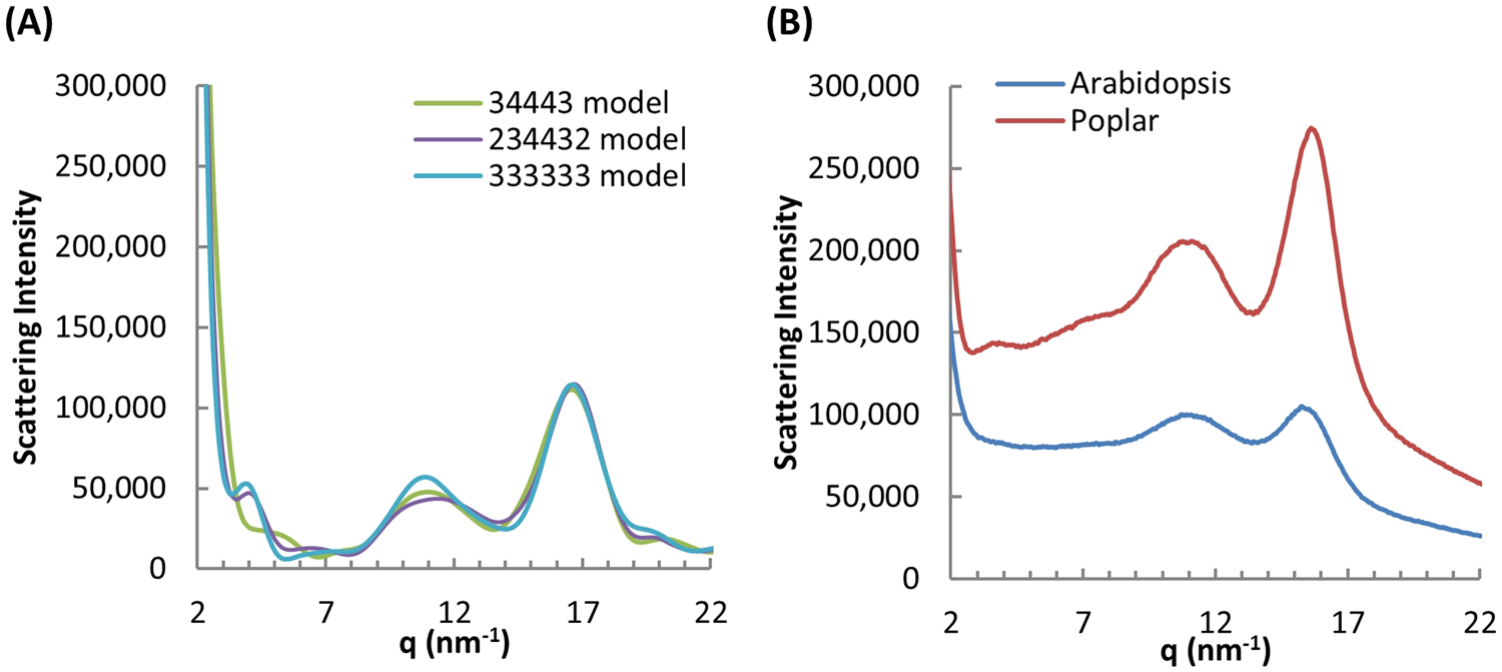
Simulated and experimental WAXS diffractograms for cellulose I. (**A**) All three CMF habits predict similar peaks near 10 to 11 and 16 nm^−1^. The 34443 model is distinct however in not producing a WAXS peak near 4 nm^−1^. (**B**) Wide-angle X-ray scattering from *Arabidopsis* and Poplar.

WAXS data were instrumental in questioning the original 36-chain CMF model, so the models in this study were used to generate synthetic diffractograms for direct comparison with experimental observations. The theoretical diffraction patterns of the three CMF habits had peaks at ~10 to 11 nm^−1^ and 16 nm^−1^ similar to the experimental data for *Arabidopsis* and *Poplar* (Fig. 2). The 34443 model is distinct however in not producing a WAXS peak near 4 nm^−1^ (Fig. 2A). In addition, the 4 nm^−1^ peak is independent of the length of the cellulose microfibril models applied in WAXSiS calculations (as shown in Supplementary Fig. 2). It is also independent of the surrounding environment (water or vacuum) of CMF, as shown in Supplementary Fig. 4. Interestingly, when comparing to experimental WAXS results, we found that the WAXS diffractogram of *Poplar* cell walls demonstrated a weak peak around 4 nm^−1^, whereas that of *Arabidopsis* did not show any peaks around that area. This is probably due to the difference in average electron density in real sample and inter-microfibril correlation. However, due to the complexity of plant cell walls, it is challenging to compare the simulation results with the experiments *in planta* especially in the q-range approaching the size of microfibrils. The peak around 4 nm^−1^ pointed us a possible way to differentiate CMF structures. However, more experiment and simulation work would be expected to further explore the origin of the peak.

## Conclusion

In conclusion, DFT calculations on the structures, energies and spectroscopic parameters of three proposed CMF habits all predict accurate structures and δ^13^C NMR values. Based comparison of sC4 and sC6 calculated versus observed δ^13^C, the 34443 model is slightly favored. Energetic considerations discount the 6 × 3 model as least favorable with periodic and molecular cluster calculations providing indistinguishable results on the 234432 and 34443 models; however, the calculated energy results favor 34443. WAXS diffractograms of the 34443 model are more consistent with observed patterns. We conclude the preponderance of evidence suggests that the 34443 habit is most probable because it cannot be eliminated from consideration based on comparisons to structural and ^13^C NMR data nor energy estimations via DFT. The most distinguishing feature is the lack of a WAXS peak near 4 nm^−1^ which is predicted to exist for the other two models considered.

## Methods

Density functional theory calculations in this study were performed with the Vienna Ab-initio Simulation Package (VASP^37–40^) and Gaussian 09^25^ codes. Initial atomic structures were based upon the experimental model of Nishiyama *et al*.^1^ as supplied by Yoshi Nishiyama. The unit cell of cellulose was doubled to include 4 glucan units along the c-dimension, and 18 polymer CMFs were constructed in Materials Studio 2016. The simulation cell was expanded in the x- and y-dimensions to allow for space between the images of the CMFs in order to minimize any self-interactions. The model structures were converted into VASP input files (POSCAR) via a perl script written by Andrei V. Bandura (Saint Petersburg State University, Russia). All atomic positions were relaxed during energy minimizations using the VASP code. The simulation cell lattice parameters were also relaxed until the c-dimension converged to a stable state in order to the cellulose chains to stretch or contract. After convergence of the chain lengths, the lattice parameters were then held constant in order to prevent artificial aggregation of the CMFs with their periodic images (i.e., contraction of the *a* and *b* cell dimensions until the CMF would sense images of itself.)

NMR calculations used the multi-standard method of^41^ with tetramethylsilane (TMS) and methanol as the primary and secondary standards^4,26,34,42,43^ in order to increase accuracy in reproducing experimental values. The modified Perdue–Wang exchange–correlation functional mPW1PW91^44^ along with the 6–31 G(d) basis set^45^ were used in the calculation of isotropic chemical shieldings for all nuclei. Only the added terminal methyl groups were relaxed in Gaussian 09 while all other atoms were fixed in the positions determined in the periodic energy minimizations performed with VASP. The 18-chain CMF structures (each chain contains 4 glucose units) were solvated in a box of TIP3P water^46^ followed the protocol detailed out in previous work^35^ using the Impact module of Maestro^47^, followed by 1000 steps of conjugate gradient minimization performed before 10 ps molecular dynamics simulations at 298 K with a 1 fs time step in the NVT ensemble using the OPLS_2005 force field^48^ keeping the atomic positions of cellulose molecules fixed. Only H_2_O molecules within 3 Å of the cellulose clusters were included for NMR calculations as it was found that H_2_O molecules further than 3 Å from the clusters had negligible effects on the calculated δ^13^C. An empirical correction of 49.5 ppm^43^ was used for the difference between the δ^13^C in methanol and TMS commonly used as an experimental ^13^C NMR standard^41^. This gives an isotropic chemical shielding of 193.0 ppm. To compute the δ^13^C for any C nucleus i in cellulose, we used: δ^13^Ci = 193.0 ppm - δ^13^Ci.

Small- and Wide-Angle X-ray Scattering curves of CMFs in solution were calculated based on explicit-solvent all-atom molecular dynamics (MD) simulations using WAXSiS^23,24^.

The 18-chain CMF structures were obtained from the periodic energy minimizations performed with VASP. Each chain contained 19 glucose residues Each CMF structure was solvated in a box of TIP3P water so that the CMF was surrounded in all directions by at least 20 Å. Short (50 ps) MD simulations were performed using AMBER^49^ and the Glycam06 carbohydrate force field^50^ at 300 K, at 1 bar, in the NPT ensemble, with 2 fs time step, 10 Å non-bonded interaction cutoff, and SHAKE-constrained hydrogen bonds. The positions of CMF were constrained using a force constant of 250 kcal/mol/Å^2^. Simulation frames are written every 0.5 ps so that the solvent configurations are uncorrelated^23^. From the MD trajectories, the theoretical scattering curves were then calculated using WAXSiS webserver. Two buffer subtraction methods have been used to calculate the scattering intensity: (i) total buffer intensity is subtracted, I(q) = I_sample_(q) − I_buffer_(q); (ii) the buffer intensity is reduced by the volume fraction v of the solute, I(q) = I_sample_(q) − (1 − v) I_buffer_(q)^23^. The two subtraction methods only slightly differ at small angles. As shown in Supplementary Fig. 3, total buffer subtraction method predicted 200 peaks at 15.8–16 nm^−1^, and buffer scattering reduced by solute volume resulted in the 200 peaks at 16.5 nm^−1^, very close to the widely accepted value for 200 of cellulose I (around 16 nm^−1^)^13,51–53^. But they highly differ at wide angles around the water scattering peak (20 nm^−1^)^23^. As shown in Supplementary Fig. 3, total buffer subtraction method resulted in negative intensities around 20 nm^−1^. However, both predicted the peak around 4 nm^−1^. The CRYSOL^54^ program was also used for calculation of theoretical diffraction patterns of the three CMF habits in water (with solvent density of 0.334 e/Å^3^) and in vacuum based on the atomic coordinate of the CMF structures obtained from the periodic energy minimizations performed with VASP.

The *Poplar* tension wood sample was obtained as described in Sawada *et al*.^55^. The bottom 1 cm section of a seven week old stem from *Arabidopsis thaliana colD* was dried at 80 °C overnight before measurement. The XRD profiles were recorded as described in Sawada *et al*.^55^.

## Electronic supplementary material

Supplementary Information

## References

[1] Nishiyama Y, Langan P, Chanzy H (2002). Crystal structure and hydrogen-bonding system in cellulose Iβ from synchrotron X-ray and neutron fiber diffraction. Journal of the American Chemical Society.

[2] Nishiyama Y, Johnson GP, French AD, Forsyth VT, Langan P (2008). Neutron crystallography, molecular dynamics, and quantum mechanics studies of the nature of hydrogen bonding in cellulose Iβ. Biomacromolecules.

[3] Nishiyama Y, Sugiyama J, Chanzy H, Langan P (2003). Crystal structure and hydrogen bonding system in cellulose Iα from synchrotron X-ray and neutron fiber diffraction. Journal of the American Chemical Society.

[4] Kubicki JD, Mohamed MN-A, Watts HD (2013). Quantum mechanical modeling of the structures, energetics and spectral properties of Iα and Iβ cellulose. Cellulose.

[5] Lee CM (2015). Hydrogen-bonding network and OH stretch vibration of cellulose: comparison of computational modeling with polarized IR and SFG spectra. The Journal of Physical Chemistry B.

[6] Cosgrove DJ (2014). Re-constructing our models of cellulose and primary cell wall assembly. Current opinion in plant biology.

[7] Jarvis MC (2018). Structure of native cellulose microfibrils, the starting point for nanocellulose manufacture. Phil. Trans. R. Soc. A.

[8] Wang, T., Yang, H., Kubicki, J. D. & Hong, M. Cellulose Structural Polymorphism in Plant Primary Cell Walls Investigated by High-Field 2D Solid-State NMR Spectroscopy and Density Functional Theory Calculations. *Biomacromolecules* (2016). 10.1021/acs.biomac.6b00441 PMC5270591 27192562

[9] Harris DM (2012). Cellulose microfibril crystallinity is reduced by mutating C-terminal transmembrane region residues CESA1A903V and CESA3T942I of cellulose synthase. Proceedings of the National Academy of Sciences.

[10] Lee CM, Kafle K, Huang S, Kim SH (2015). Multimodal broadband vibrational sum frequency generation (MM-BB-V-SFG) spectrometer and microscope. The Journal of Physical Chemistry B.

[11] Herth W, Weber G (1984). Occurrence of the putative cellulose-synthesizing “rosettes” in the plasma membrane of Glycine max suspension culture cells. Naturwissenschaften.

[12] Newman RH, Hill SJ, Harris PJ (2013). Wide-angle x-ray scattering and solid-state nuclear magnetic resonance data combined to test models for cellulose microfibrils in mung bean cell walls. Plant physiology.

[13] Fernandes AN (2011). Nanostructure of cellulose microfibrils in spruce wood. Proceedings of the National Academy of Sciences.

[14] Nixon BT (2016). Comparative structural and computational analysis supports eighteen cellulose synthases in the plant cellulose synthesis complex. Scientific reports.

[15] Hill JL, Hammudi MB, Tien M (2014). The Arabidopsis cellulose synthase complex: a proposed hexamer of CESA trimers in an equimolar stoichiometry. The Plant Cell.

[16] Vandavasi VG (2016). A structural study of CESA1 catalytic domain of Arabidopsis cellulose synthesis complex: evidence for CESA trimers. Plant physiology.

[17] Yang H, Zimmer J, Yingling YG, Kubicki JD (2015). How Cellulose Elongates · A QM/MM Study of the Molecular Mechanism of Cellulose Polymerization in Bacterial CESA. The Journal of Physical Chemistry B.

[18] Sethaphong L (2013). Tertiary model of a plant cellulose synthase. Proceedings of the National Academy of Sciences.

[19] Morgan JL, Strumillo J, Zimmer J (2013). Crystallographic snapshot of cellulose synthesis and membrane translocation. Nature.

[20] Li Y, Lin M, Davenport JW (2011). Ab initio studies of cellulose I: crystal structure, intermolecular forces, and interactions with water. The Journal of Physical Chemistry C.

[21] Bučko T, Tunega D, Ángyán JG, Hafner J (2011). Ab initio study of structure and interconversion of native cellulose phases. The Journal of Physical Chemistry A.

[22] Goldberg RN (2015). A thermodynamic investigation of the cellulose allomorphs: Cellulose (am), cellulose Iβ (cr), cellulose II (cr), and cellulose III (cr). The Journal of Chemical Thermodynamics.

[23] Knight CJ, Hub JS (2015). WAXSiS: a web server for the calculation of SAXS/WAXS curves based on explicit-solvent molecular dynamics. Nucleic acids research.

[24] Chen P, Hub JS (2014). Validating solution ensembles from molecular dynamics simulation by wide-angle X-ray scattering data. Biophysical journal.

[25] Frisch, M. *et al*. Gaussian 09, revision B. 01. Gaussian, Inc., Wallingford, CT (2010).

[26] Watts HD, Mohamed MNA, Kubicki JDA (2014). DFT study of vibrational frequencies and 13C NMR chemical shifts of model cellulosic fragments as a function of size. Cellulose.

[27] Grimme S, Hansen A, Brandenburg JG, Bannwarth C (2016). Dispersion-corrected mean-field electronic structure methods. Chemical reviews.

[28] Grimme S, Ehrlich S, Goerigk L (2011). Effect of the damping function in dispersion corrected density functional theory. Journal of computational chemistry.

[29] Perdew JP, Wang Y (1992). Accurate and simple analytic representation of the electron-gas correlation energy. Physical Review B.

[30] Zhao Y, Schultz NE, Truhlar DG (2006). Design of density functionals by combining the method of constraint satisfaction with parametrization for thermochemistry, thermochemical kinetics, and noncovalent interactions. Journal of Chemical Theory and Computation.

[31] Becke AD (1993). A new mixing of Hartree–Fock and local density‐functional theories. The Journal of chemical physics.

[32] Lee C, Yang W, Parr RG (1988). Development of the Colle-Salvetti correlation-energy formula into a functional of the electron density. Physical review B.

[33] Cances E, Mennucci B, Tomasi J (1997). A new integral equation formalism for the polarizable continuum model: Theoretical background and applications to isotropic and anisotropic dielectrics. The Journal of chemical physics.

[34] Kubicki JD, Watts HD, Zhao Z, Zhong L (2014). Quantum mechanical calculations on cellulose–water interactions: structures, energetics, vibrational frequencies and NMR chemical shifts for surfaces of Iα and Iβ cellulose. Cellulose.

[35] Yang, H. *et al*. Structural factors affecting 13C NMR chemical shifts of cellulose: a computational study. *Cellulose*, 1–14 (2017).

[36] Wang T, Hong M (2015). Solid-state NMR investigations of cellulose structure and interactions with matrix polysaccharides in plant primary cell walls. Journal of experimental botany.

[37] Kresse G, Furthmüller J (1996). Efficient iterative schemes for ab initio total-energy calculations using a plane-wave basis set. Physical review B.

[38] Kresse G, Furthmüller J, Hafner J (1994). Theory of the crystal structures of selenium and tellurium: the effect of generalized-gradient corrections to the local-density approximation. Physical Review B.

[39] Kresse G, Hafner J (1993). Ab initio molecular dynamics for open-shell transition metals. Physical Review B.

[40] Kresse G, Hafner J (1994). Ab initio molecular-dynamics simulation of the liquid-metal–amorphous-semiconductor transition in germanium. Physical Review B.

[41] Sarotti AM, Pellegrinet SC (2009). A multi-standard approach for GIAO 13C NMR calculations. The Journal of organic chemistry.

[42] Watts HD, Mohamed MNA, Kubicki JD (2011). Comparison of multistandard and TMS-standard calculated NMR shifts for coniferyl alcohol and application of the multistandard method to lignin dimers. The Journal of Physical Chemistry B.

[43] Gottlieb HE, Kotlyar V, Nudelman A (1997). NMR chemical shifts of common laboratory solvents as trace impurities. The Journal of organic chemistry.

[44] Adamo C, Barone V (1998). Exchange functionals with improved long-range behavior and adiabatic connection methods without adjustable parameters: The mPW and mPW1PW models. The Journal of Chemical Physics.

[45] Rassolov VA, Ratner MA, Pople JA, Redfern PC, Curtiss LA (2001). 6‐31G* basis set for third‐row atoms. Journal of Computational Chemistry.

[46] Jorgensen WL, Chandrasekhar J, Madura JD, Impey RW, Klein ML (1983). Comparison of simple potential functions for simulating liquid water. The Journal of chemical physics.

[47] Schrödinger Release 2014-1: Maestro, version 9.7 (2014).

[48] Banks JL (2005). Integrated modeling program, applied chemical theory (IMPACT). Journal of computational chemistry.

[49] Case, D. A. *et al*. Amber **14** (2014).

[50] Kirschner KN (2008). GLYCAM06: a generalizable biomolecular force field. Carbohydrates. Journal of computational chemistry.

[51] Thomas LH (2015). Diffraction evidence for the structure of cellulose microfibrils in bamboo, a model for grass and cereal celluloses. BMC plant biology.

[52] Crawshaw J, Bras W, Mant G, Cameron R (2002). Simultaneous SAXS and WAXS investigations of changes in native cellulose fiber microstructure on swelling in aqueous sodium hydroxide. Journal of applied polymer science.

[53] Gubitosi M (2017). Stable, metastable and unstable cellulose solutions. Royal Society open science.

[54] Svergun D, Barberato C, Koch MH (1995). CRYSOL–a program to evaluate X-ray solution scattering of biological macromolecules from atomic coordinates. Journal of applied crystallography.

[55] Sawada D (2018). Tension wood structure and morphology conducive for better enzymatic digestion. Biotechnology for biofuels.

