# Supplementary material for: The Shape of Native Plant Cellulose Microfibrils
Source: Sci Rep. 2018 Sep 18;8:13983. doi: 10.1038/s41598-018-32211-w (PMC6143632; doi:10.1038/s41598-018-32211-w)
Supplement: Supplementary file 1 — Supplementary Information [file 41598_2018_32211_MOESM1_ESM.docx]

**Title:** The Shape of Native Plant Cellulose Microfibrils

**Authors:** James D. Kubicki^1^*, Hui Yang^2^, Daisuke Sawada^3^, Hugh O’Neill^4^, Daniel Oehme^1^, Daniel Cosgrove^2^

**Affiliations:**

^1^ Department of Geological Sciences, University of Texas at El Paso, El Paso, TX, USA.

^2^ Department of Biology, The Pennsylvania State University, University Park, PA, USA.

^3^ Department of Bioproducts and Biosystems, School of Chemical Engineering, Aalto University, Espoo, Finland.

^4^ Neutron Scattering Division, Oak Ridge National Laboratory, Oak Ridge, USA.

*Correspondence to: [jdkubicki@utep.edu](mailto:jdkubicki@utep.edu)

Supplementary Materials:


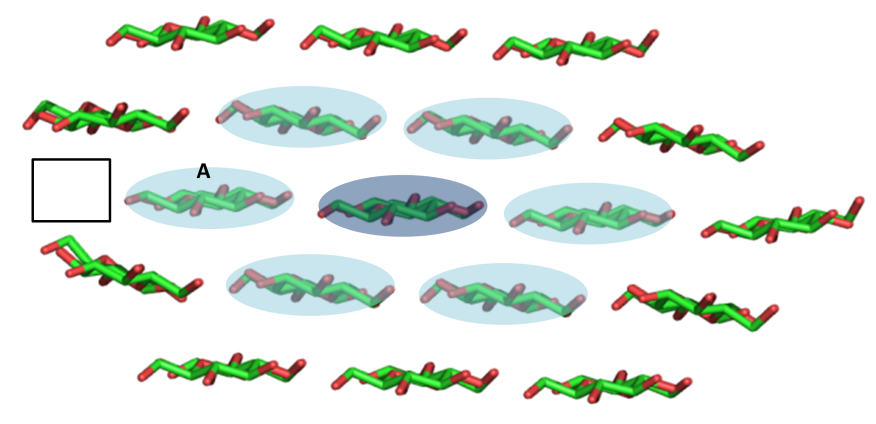


**Supplementary Figure 1.** The 34443 CMF model. The core domain is shaded in darker blue. The surface-bound fraction is shaded in lighter blue color. The cavity beside the cellulose chain A is indicated by a rectangular box.

**Supplementary Figure 2.** The peaks near 4 nm^-1^ are independent upon the length of the model. WAXS diffractograms were calculated using CRYSOL. (xxxxx-Ymer: xxxxx stands for the specific arrangement of cellulose polymer, e.g. 333333, 34443, and 234432. Ymer represents that each cellulose chain has Y glucose units.)

**Supplementary Figure 3.** Both buffer subtraction methods predicted the WAXS peak near 4 nm^-1^ for 234432 and 333333 models. Left: Buffer scattering reduced by solute volume, Right: Total buffer scattering subtracted.

**Supplementary Figure 4.** The peak near 4 nm^-1^ is independent on the surrounding environment. Left: In pure water, Right: In vacuum.

**Supplementary Table 1.** Calculated δ^13^C (ppm) for surficial cellulose chains in water

|  | **C4-H4 pointing out** | | | | | | **C4-H4 pointing in** | | | | | |
| --- | --- | --- | --- | --- | --- | --- | --- | --- | --- | --- | --- | --- |
|  | **C1** | **C2** | **C3** | **C4** | **C5** | **C6** | **C1** | **C2** | **C3** | **C4** | **C5** | **C6** |
| **333333** | 104.9 | 72.0 | 72.7 | 87.5 | 72.3 | 65.9 | 104.8 | 70.7 | 73.2 | 85.8 | 71.5 | 66.3 |
| **Std dev** | 0.9 | 1.6 | 1.8 | 2.4 | 1.4 | 1.2 | 2.0 | 0.6 | 1.3 | 1.3 | 1.4 | 1.4 |
| **34443** | 104.6 | 72.0 | 72.2 | 87.2 | 71.6 | 66.3 | 104.8 | 70.1 | 73.1 | 85.4 | 72.5 | 65.3 |
| **Std dev** | 0.8 | 1.2 | 1.0 | 1.9 | 1.5 | 1.1 | 1.3 | 1.1 | 1.3 | 0.9 | 1.1 | 0.6 |
| **234432** | 104.7 | 71.2 | 73.0 | 86.7 | 71.6 | 65.9 | 104.4 | 70.6 | 72.3 | 85.5 | 72.4 | 66.4 |
| **Std dev** | 1.1 | 1.5 | 0.9 | 0.5 | 1.1 | 1.1 | 0.8 | 0.8 | 0.9 | 0.9 | 1.3 | 1.0 |

**Supplementary Table 2**. NMR chemical shifts (ppm) of cellulose chain A are similar to an interior center chain. All NMR chemical shifts were calculated in vacuum.

|  |  | **C1** | **C2** | **C3** | **C4** | **C5** | **C6** |
| --- | --- | --- | --- | --- | --- | --- | --- |
| **Interior** | **Origin** | 105.4 | 70.3 | 73.2 | 85.7 | 73.0 | 65.5 |
|  | **Center** | 102.5 | 69.8 | 73.9 | 84.7 | 70.9 | 65.9 |
| **Surface** | **C4-H4 pointing out** | 104.9 | 72.1 | 73.8 | 90.2 | 70.6 | 66.4 |
|  | **C4-H4 pointing in** | 106.6 | 68.7 | 75.8 | 84.9 | 73.3 | 65.6 |
| **Cellulose chain A** | | 102.0 | 70.5 | 73.9 | 84.3 | 70.1 | 64.9 |
|  |  | 102.4 | 69.3 | 72.7 | 86.0 | 71.0 | 66.5 |
| **Core domain** | | 102.4 | 70.6 | 74.2 | 84.9 | 71.1 | 65.5 |
|  |  | 102.9 | 68.8 | 73.8 | 84.9 | 70.9 | 66.2 |
